# Carney complex with multiple breast tumours including breast cancer: a case report

**DOI:** 10.1093/omcr/omac063

**Published:** 2022-06-23

**Authors:** Akihiro Fujimoto, Ayaka Sakakibara, Yoshiki Numajiri, Kazuo Matsuura, Tomonori Kawasaki, Akihiko Osaki, Toshiaki Saeki

**Affiliations:** Breast Oncology Service, Saitama Medical University International Medical Center, Hidaka, Saitama JP 350-1298, Japan; Breast Oncology Service, Saitama Medical University International Medical Center, Hidaka, Saitama JP 350-1298, Japan; Department of Digestive Surgery, IMS Miyoshi General Hospital, Miyoshi, Saitama JP 354-0041, Japan; Breast Oncology Service, Saitama Medical University International Medical Center, Hidaka, Saitama JP 350-1298, Japan; Department of Pathology, Saitama Medical University International Medical Center, Hidaka, Saitama JP 350-1298, Japan; Breast Oncology Service, Saitama Medical University International Medical Center, Hidaka, Saitama JP 350-1298, Japan; Breast Oncology Service, Saitama Medical University International Medical Center, Hidaka, Saitama JP 350-1298, Japan

## Abstract

Carney complex (CNC) is a rare multiple tumour syndrome characterized by cutaneous pigmented lesions, myxoma and endocrine tumours, among others, and is inherited as an autosomal dominant trait. Protein kinase cAMP-dependent type I regulatory subunit alpha (*PRKAR1A*) is known to be the responsible gene. Breast myxomatosis and ductal adenoma, which are regarded as benign, are well-known mammary lesions of CNC and are included in the main diagnostic criteria. In this case, a 59-year-old woman with repeated cardiac myxoma was diagnosed with CNC with *PRKAR1A* mutation. She also had three multiple breast tumours bilaterally: breast cancer, adenomyoepithelioma and intraductal papilloma. In mammary lesions of CNC, attention should be paid to benign lesions, such as breast myxomatosis or ductal adenomas, and the development of breast cancer or breast tumours with malignant potential. Mammary lesions should be aggressively scrutinized and considered for resection, as required.

## INTRODUCTION

Carney complex (CNC) is a multiple tumour syndrome characterized by cutaneous pigmented lesions, myxoma mainly on the skin/heart/breast and endocrine or neural tumours, among others. CNC is inherited as an autosomal dominant trait and caused by inactivating mutations of the protein kinase cAMP-dependent type I regulatory subunit alpha (*PRKAR1A*) gene. In families affected by CNC, 62% had a detectable *PRKAR1A* mutation [1]. *De novo* mutations in *PRKAR1A* have been detected in about one-third of CNC cases [2]. For mammary gland lesions, breast myxomatosis and breast ductal adenomas are included in the CNC’s main diagnostic criteria; however, the involvement of breast cancer and other breast tumours is currently not clearly known. Herein, we encountered a CNC case with multiple breast tumours that were not myxomatosis or breast ductal adenoma but breast cancer, adenomyoepithelioma (AME) and intraductal papilloma (IDP).

## CASE REPORT

**Figure 1:**
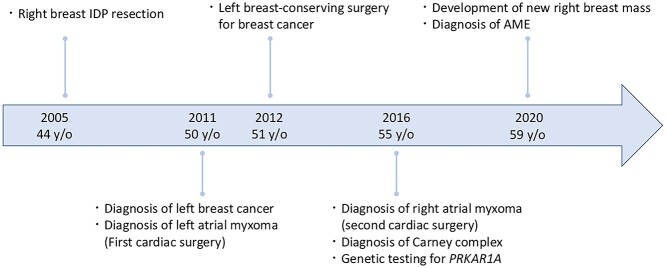
Important timelines.

**Figure 2:**
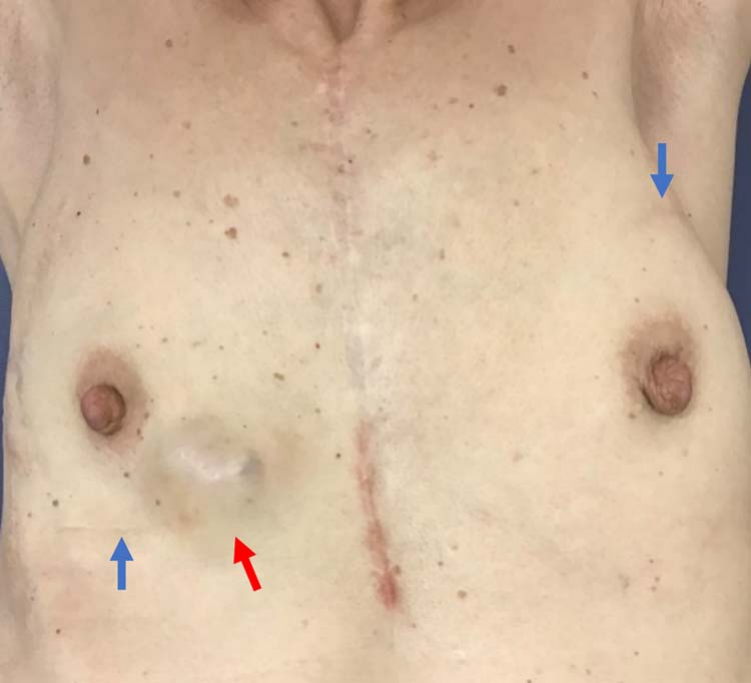
Physical findings in 2020. Many brown spotty skin pigmentations on the anterior chest. Palpable breast mass in the right breast (red arrow). Surgical scars on the bilateral breasts (blue arrows).

**Figure 3:**
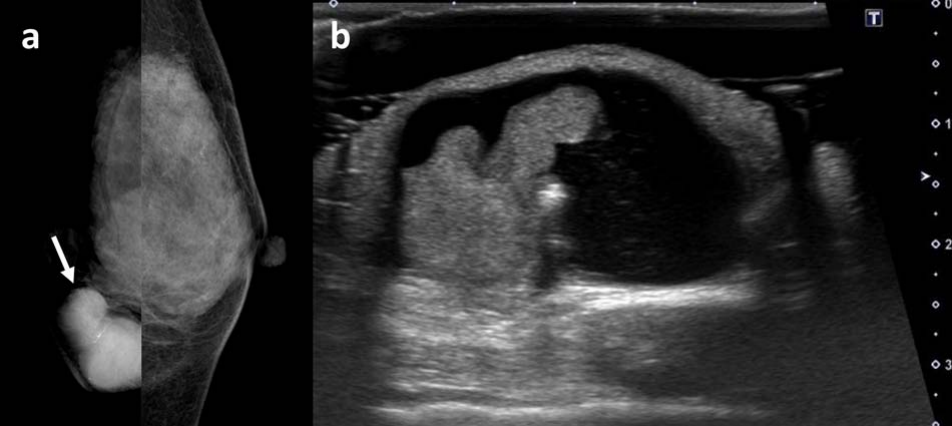
Clinical images of the right breast tumour in 2020. (**a**) Mammography showing a high-density mass with circumscribed margins (arrow). (**b**) Ultrasonography showing a 38-mm complicated cyst.

**Figure 4:**
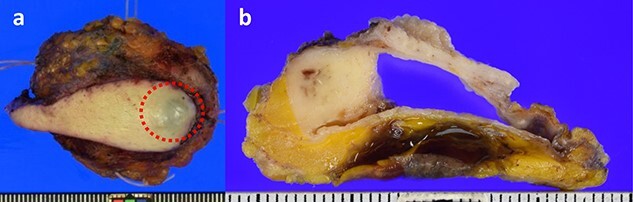
Macroscopic findings of the specimen. (**a**) Tumour location is in the red circle. (**b**) Cut surface of the excised specimen.

**Figure 5:**
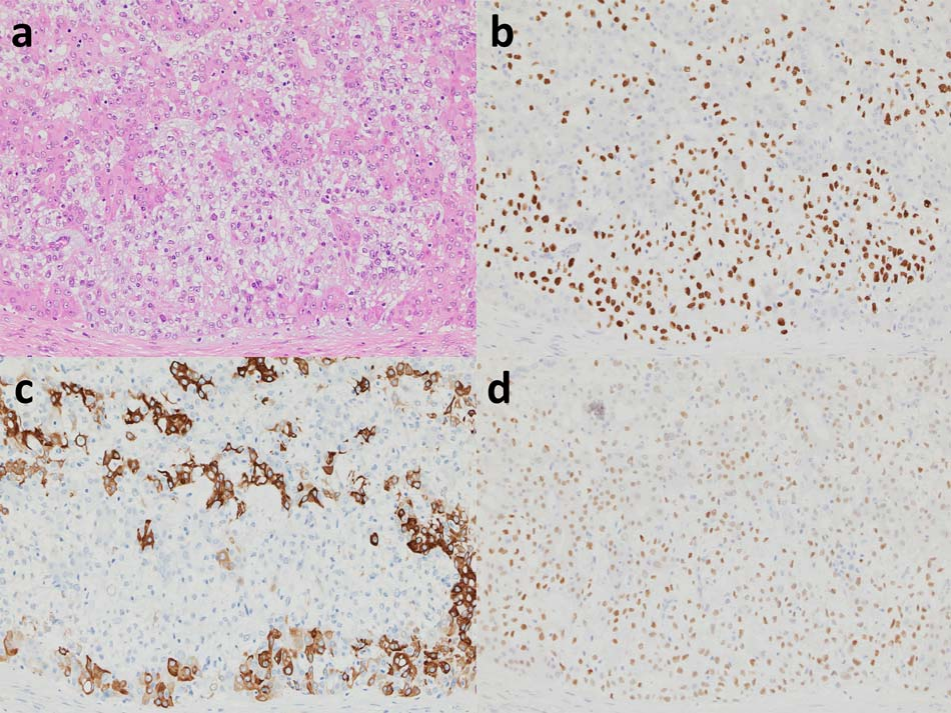
Histopathological findings of AME. (**a**) The tumour comprises a mosaic admixture of eosinophilic epithelial cell components and clear myoepithelial cell elements (haematoxyin and eosin, ×200). (**b**) Immunohistochemically, p63 is positive in myoepithelial cells (×200). (**c**) Cytokeratin 14 is antithetically reactive in epithelial cells (×200). (**d**) Oestrogen receptor shows diffuse and relatively weak immuno-expressions in tumour cells (×200).

A 59-year-old woman presented with a mass in the breast. Family history revealed that her father had skin cancer in his 60s, and her paternal aunt had osteosarcoma in her 40s. Her medical history was as follows (Fig. 1): in 2005, she underwent a right breast tumourectomy and was diagnosed with IDP. In 2011, left breast cancer was detected, and preoperative screening echocardiography coincidentally revealed left atrial myxoma; cardiac surgery was performed to remove the myxoma. In 2012, left breast-conserving surgery and sentinel lymph node biopsy were performed; infiltrating elements of histological grade 1, measuring 13 mm, were observed in the lesion. On immunohistochemical examination, neoplastic cells were positive for oestrogen receptor and progesterone receptor (total Allred scores: 8 and 8, respectively). The human epidermal growth factor receptor 2 score was 1+ in invasive neoplastic cells. The final diagnosis was stage IA invasive ductal carcinoma (pT1cN0M0), luminal subtype. Tamoxifen was administered as adjuvant hormone therapy based on pathological findings. Additionally, the patient underwent whole left breast radiotherapy (42.56 Gy). In 2016, she was diagnosed with right atrial myxoma and underwent cardiac surgery again. CNC was suspected because of repeated cardiac myxoma, and she was confirmed to meet the CNC diagnostic criteria. Moreover, genetic testing revealed a *PRKAR1A* pathogenic variant. Conversely, genetic testing to identify germline mutations in other genes, such as *BRCA1/2*, was not performed. Additionally, somatic gene mutations in breast cancer were not investigated. In 2020, she developed a new right breast mass (Fig. 2). Physical examination revealed ocular conjunctiva, and pigmentation was observed in the right eye; brown spotty skin pigmentation was observed on the lips, surrounding area and anterior chest (Fig. 2). The right breast mass was described as a complicated cyst on mammography and ultrasonography (Fig. 3); needle biopsy confirmed atypical ductal hyperplasia. Therefore, a right breast tumourectomy was performed (Fig. 4). Based on histopathological findings (Fig. 5), the final diagnosis was AME of the breast. Since the tumour was resected, the post-operative policy was regular follow-up without additional treatment. Currently, no apparent recurrence or metastasis of any breast tumour has been observed.

## DISCUSSION

This case report highlights two crucial clinical findings. First, CNC could be relevant to the bilateral development of multiple breast tumours, other than breast myxomatosis and breast ductal adenoma. Second, in mammary lesions of CNC, attention should be paid to the occurrence of breast cancer or breast tumours with malignant potential.

The most common CNC-associated mammary tumour is breast myxomatosis, which occurs in ~20% of female patients after puberty and is often bilateral [3]. Second, ductal adenoma also often occurs, with a frequency of ~3% in female patients [4]. Both are considered benign lesions; reports of malignant transformation in patients are unavailable [5]. However, in a report investigating 338 patients with CNC, only one patient (0.3%) had breast cancer [6]. To the best of our knowledge, this is the first report describing a CNC case with breast cancer, AME and IDP.

Breast lesions commonly found in patients with CNCs are generally benign and unlikely to significantly impact prognosis. However, *PRKAR1A* is associated with breast cancer [7] because the protein kinase A signal is activated by *PRKAR1A* loss and promotes mammary tissue carcinogenesis [8]. Therefore, attention should be paid to breast cancer complications, especially in patients with CNC with *PRKAR1A* mutation, as in this case. Conversely, AME is a rare tumour characterized by biphasic proliferation of ductal epithelium and myoepithelial cells [9]. AME is associated with a spectrum of diseases, ranging from benign to malignant tumours [10]; hence, surgical resection is often considered. Appropriate tissue biopsy is important to ensure that these lesions are not overlooked.

This is an unusual case of three different breast tumours—breast cancer, AME and IDP. The history of these breast tumours alone does not suggest possible CNC. However, if attention has been paid to the distinctive pigmentation of the anterior chest and cardiac myxoma, even a breast surgeon may have been able to consider any genetic disease. The possibility of a genetic disorder should be considered, even if it is unlikely to be positive.

Regarding breast surveillance in diagnosed CNC cases, more intensive follow-up with contrast-enhanced magnetic resonance imaging with mammography and ultrasonography may be recommended for preservation and early detection of similar cases, following the hereditary breast–ovarian cancer syndrome. Moreover, a family history of carcinoma, particularly of the thyroid, colon, pancreas and ovaries and other multiple benign or malignant tumours, is a finding suggestive or possibly associated with CNC [6]; therefore, for surveillance of organs for carcinogenesis, other than the breast, we should particularly focus on these organs.

In conclusion, multiple breast tumours can develop bilaterally in CNC, including breast cancer, even if breast myxomatosis and breast ductal adenoma do not exist. Biopsy of breast lesions may be postponed or breast lesions may be left untreated even if they are pathologically diagnosed; however, a malignant breast tumour can coexist, as in this case. Therefore, actively scrutinizing breast lesions is important. Further studies investigating the relationship between CNC and breast tumours, including breast cancer, are warranted.
